# Critical Role of 3D ultrasound in the diagnosis and management of Robert's uterus: a single-centre case series and a review

**DOI:** 10.52054/FVVO.13.1.008

**Published:** 2021-03-31

**Authors:** M Deenadayal, V Günther, I Alkatout, D Freytag, A Deenadayal - Mettler, A Deenadayal Tolani, R Sinha, L Mettler

**Affiliations:** Mamata Fertility Hospital, 9-1-192, St Marys Rd, Telangana 500003, Hyderabad, India; Department of Obstetrics and Gynecology, University Hospitals Schleswig-Holstein, Campus Kiel, Arnold-Heller-Str. 3, 24105 Kiel, Germany; Apollo Health City, Gynaecology, Road No 72, Hyderabad, Telangana 500033, Hyderabad, India

**Keywords:** Robert's uterus, congenital uterine anomalies, laparoscopy, hysteroscopy

## Abstract

A septate uterus with a non-communicating hemicavity was first described by Robert in 1969/70 as a specific malformation of the uterus. The condition is commonly associated with a blind uterine hemicavity, unilateral haematometra, a contralateral unicornuate uterine cavity and a normal external uterine fundus. The main symptoms are repetitive attacks of pain at four-weekly intervals around menarche, repeated dysmenorrhea, recurrent pregnancy loss and infertility.

In this report, we review the disease, its diagnosis and treatment, and describe five cases of Robert's uterus. Three dimensional (3D) ultrasound (US) imaging was performed by the transvaginal route in four cases. In the fifth case of a 13-year-old girl, we avoided the vaginal route and magnetic resonance imaging (MRI) and 3D transrectal US yielded the correct diagnosis.

The following treatment procedures were undertaken: laparoscopic endometrectomy, hysteroscopic septum resection, laparoscopic uterine hemicavity resection and total laparoscopic hysterectomy (TLH). The diagnosis and optimum treatment of Robert's uterus remains difficult for clinicians because of its rarity. A detailed and careful assessment by 3D US should be performed, followed by hysteroscopy in combination with laparoscopy, to confirm the diagnosis.

## Introduction

Female genital malformations are defined as deviations from normal anatomy that could impair the reproductive potential of a woman or, in complex cases such as obstructive anomalies, a woman’s health. The malformations arise embryologically from the failure of Müllerian duct formation, canalisation, fusion or absorption. The anomalies are either isolated defects or occur in combination with conditions in various parts of the female genital tract. The latter results in so-called complex anomalies.

The overall prevalence of uterine anomalies is 0.5%. Awareness of these conditions is extremely important in order to avoid misdiagnosis and inappropriate management (Acien et al., 2004; Gupta et al., 2007; Deutch and Abuhamad, 2008).

Pelvic pain, prolonged or abnormal bleeding at the time of menarche, recurrent pregnancy loss, or preterm delivery are symptoms of congenital uterine anomalies (CUAs). Some CUAs may be suspected because of associated findings on physical examination, such as a longitudinal vaginal septum. Others may be detected when imaging studies are performed to evaluate patients with severe menstrual pain, cyclical pain without bleeding in young girls, infertility, symptoms related to non-reproductive organ systems, or trauma.

Classification systems are useful because they permit grouping of similar anomalies. Conclusions can be derived on the basis of several cases rather than individual case reports. The classification, diagnosis, and clinical manifestations of major congenital anomalies of the corpus (septate, unicornuate, bicornuate, or didelphys uterus), along with their associated cervical and vaginal anomalies have been discussed in the past and were summarised in the American Fertility Society Classification of 1988, by Grimbizis et al. in 2013 and 2016, and by Di Spiezio Sardo et al. in 2015 (Grimbizis et al., 2013; Di Spiezio Sardo et al. 2015; Grimbizis et al., 2016). A few comprehensive evaluations of CAUs have also been published.

The incidence of Robert's uterus is rare with only a few cases reported so far (Li et al., 2015; Di Spiezio Sardo et al., 2016). It is characterised by a septate uterus with a non-communicating blind uterine hemicavity, a contralateral unicornuate uterine cavity communicating with the cervix, and an external uterine fundus of normal shape and was first published by Robert in 1969/1970. As the condition is rare, it is not as well characterised as other uterine anomalies. Moreover, the number of cases reported in the published literature is scarce. Knowledge of this condition is extremely important in order to avoid misdiagnosis and inappropriate management. A Robert's uterus identified early can be managed by minimally invasive procedures. Failure to identify the condition may lead to adnexal lesions or endometriomas (Ludwin et al., 2013; Ludwin et al., 2014; Ludwin et al., 2017; Ludwin et al., 2018).

The origin of the defect may be a segmental agenesis of the isthmus with a persistent septum between the upper portions of the Müllerian ducts. The first symptoms of this condition are usually experienced after menarche. The obstructed unicornuate cavity has a functional endometrium which results in the retention of menstrual blood and can cause a haematometra or even a haematosalpinx. This is followed by cyclic dysmenorrhea. Reflux of the retained menstrual blood into the peritoneal cavity may be an aetiological factor for endometriosis. The second uterine cavity communicates with the single cervix and is responsible for menstrual flow.

The ESHRE-ESGE classification classified the Robert`s uterus as a Class U2bC3V0 anomaly or a complete uterus with partial cervical aplasia. Robert's uterus is also known as an asymmetric septate uterus with characteristics of a complete uterine septum, asymmetrically separating the endometrial cavity from the non-communicating hemi-uterus due to obstruction caused by the septum. With some exceptions, laparoscopy combined with hysteroscopy is the gold standard for confirming the diagnosis of Robert's uterus. The shape of the uterus may be normal on laparoscopy. There may be a normal external uterine fundal contour or a slight hollow or protrusion, with or without haematometra and haematosalpinx. On hysteroscopy the uterus has only one ostium of the fallopian tube.

In the following cases, we present five different patients with Robert`s uterus in order to show the wide variety of symptoms, the heterogeneity of the patient population, and the different diagnostic and treatment options available.

## Methods

Three dimensional (3D) ultrasound (US) imaging: In the present study, we used an advanced 3D US imaging technique for the diagnosis and postoperative screening of Robert's uterus. In our opinion, US imaging is superior to magnetic resonance imaging (MRI) screening; the latter is expensive and less widely available. To our knowledge, this is the first report of five cases of Robert's uterus from a single centre over a 12 month period.

We used the Voluson E10BT19 device transabdominally, combined with transvaginal US.

## Case series highlighting different surgical treatment options

### 

We performed an endometrectomy under laparoscopic control with restitution of uterine integrity in a 13-year-old girl, one hysteroscopic septum resection, one laparoscopic uterine resection of a hemicavity, and one hysterectomy. The fifth patient had recurrent miscarriage and was scheduled to undergo a hysteroscopic septum resection, but became pregnant again before the treatment could be performed.

In the following, we report in detail five cases of Robert's uterus, their 3D US diagnosis, and their subsequent hysteroscopic and laparoscopic treatments (Table I).

**Table I:** Patients ´data.

Case	Age (years)	Key information	Thickness of septum (mm)	Vascularity score	Associated findings	Surgery
1	13	Dysmenorrhea	13.5	2		Endometrectomy of the blind cavity and closure of the cavity.
2	25	Primary infertility, Dysmenorrhea	4.1	1	Adenomyosis, endometrioma	Hysteroscopic septal resection.
3	36	2 full term LSCS, Dysmenorrhea	27	2		Laparoscopic excision of the blind horn.
4	39	2 live children	10	2	Adenomyosis, recurrent Grade 4 endometriosis	Hysterectomy with unilateral salpingo- oophorectomy (recurrent endometrioma)
5	28	3 abortions at 16 weeks	3	1		Patient conceived during the investigations.

The key factors that play a role in deciding the treatment include age of the patient, concurrent adenomyosis/ endometriosis and the desire to retain childbearing ability. Using US we diagnose the Robert`s uterus itself and also assess the thickness and vascularity of the septum and the thickness of the uterine wall.

### Case 1: Teenage girl with dysmenorrhea

A 13-year-old girl presented with incapacitating dysmenorrhea starting three days after commencement of her menstrual cycle, associated with pain radiating to her back, vomiting and constipation.

**Figure 1:**
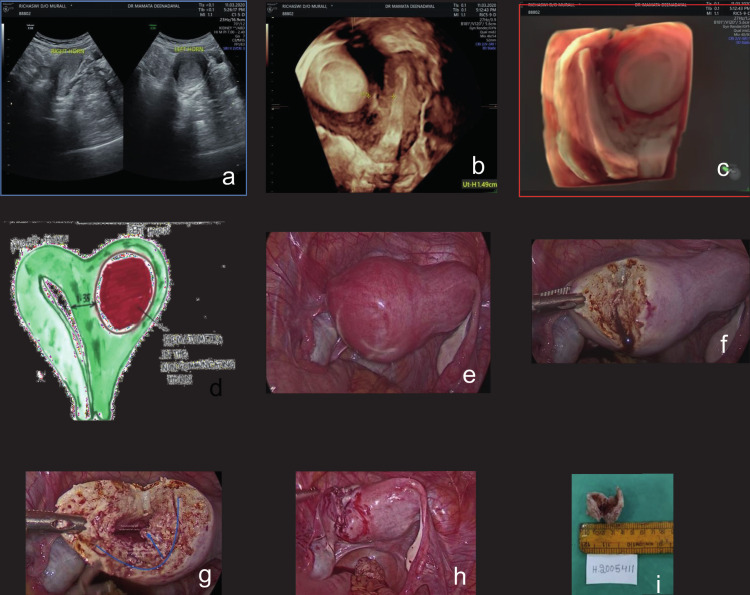
— (a) 2D US suggested a haematometra on the left side and a right uterine hemicavity, with an endometrium of 10 mm (b) Transrectal US with a C1-5D probe confirmed a haematometra of 5x5 cm on the left side, and a right hemicavity with an endometrium of 10 mm (c) The transrectal scan with a RIC-9D probe, a 3D-rendered view and HD live confirmed the presence of a haematometra in the left horn, a unicornuate-like right hemicavity and a septum of 1.49 cm dividing the two cavities (d) Explanatory line drawing of Robert's uterus (e) Laparoscopic view of the uterus (f-h) Surgical endometrectomy and myometrial reconstruction of the right uterine hemicavity (i) Excised specimen.

As the transabdominal two dimensional (2D) US provided no clear information (Fig. 1a) and the patient was not sexually active, we had to consider other imaging options such as MRI and rectal US imaging. The latter was explained to the patient and her parents, who consented to the procedure. Transrectal US was performed with volume options, contrast imaging in the multiplanar mode, and the HD live rendering mode, which identified a blind uterine horn with unilateral haematometra and a contralateral unicornuate uterine cavity. Both kidneys were normal.

Transrectal US with a C1-5D probe also revealed a haematometra measuring 5x5 cm on the left side, and a right horn with an endometrium of 10 mm, type III (Fig. 1b).

Transrectal US investigation with a RIC-9D probe, 3D rendering, and HD live confirmed the presence of haematometra in the left hemicavity, a unicornuate-like right hemicavity, and a septum of 1.49 cm thickness dividing the two cavities. The outer contour of the uterus was normal, thus confirming the diagnosis of Robert's uterus (Fig. 1c).

The images were useful in counselling the patient regarding the surgical approach. The exact anomaly was illustrated in a line drawing and enabled clear communication between the ultrasonographer, the surgeon, and the patient (Fig. 1d).

As the thickness of the septum between the two cavities was between 1.6 cm and 1.35 cm in the longitudinal aspect and was well vascularised (colour score 2), we decided to perform a laparoscopic endometrectomy. An initial diagnostic hysteroscopy revealed a single right hemicavity with a right ostium. The laparoscopic view is shown in Figure 1e.

The treatment was performed as a laparoscopic procedure. An incision was made on the fundus (Fig. 1f), and the endometrial cavity along with haematometra was identified (Fig. 1g).

The excision was performed with a margin of 2-3 mm of myometrium on all sides. The myometrium was subsequently reconstructed on the left side of the uterus (Fig. 1h). The excised specimen is shown in Figure 1i. We selected this procedure because the septum that separated the two cavities was thick; hysteroscopic resection would have been difficult in this setting. Excision of the functioning endometrial cavity relieved the patient of her intractable dysmenorrhea. The resultant single right hemicavity of the uterus is expected to be sufficient for obstetric purposes in the future.

The procedure was selected because the patient was young and we wished to avoid an extensive operative hysteroscopy due to the risks of perforation and additional damage. Alternatively, we could have performed stepwise hysteroscopy-guided opening of the septum to release the haematometra and eventually transform both cavities into a single cavity. Post-operatively no pain was reported suggesting a good clinical outcome.

### Case 2: Primary infertility with endometriosis

A 25-year-old patient with primary infertility and regular menstrual cycles presented with severe dysmenorrhea, which persisted for seven days after the end of her period. A transverse 2D image by transvaginal US with a RIC5-9D volume probe revealed a haematometra in the left hemicavity of the uterus. The right hemicavity showed a unicornuate- like cavity and adenomyosis with myometrial cysts.

**Figure 2:**
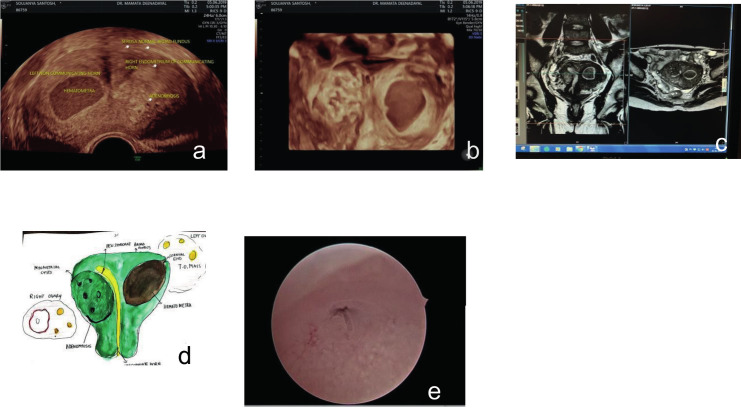
— (a) 2D US image with a transvaginal RIC 5-9D volume probe of the left horn haematometra and a right-sided unicornuate cavity (b) 3D-rendered view showing a 4.1-mm-thick septum and a normal outer contour of uterus (c) MRI image (d) Explanatory drawing (e) Hysteroscopic view of the right tubal ostium.

The outer contour of the transverse section of the uterus appeared normal. The left ovary revealed an endometrioma of 3 cm adherent to the fundus of the uterus (Fig. 2a).

The 3D rendered view demonstrated a 4.1-mm- thick septum with no vascularity (colour score 1). The outer contour of the uterus appeared normal on the coronal section (Fig. 2b). An MRI image confirmed the 3D-rendered image and provided no additional information (Fig. 2c).

The explanatory drawing served as a basis for explaining the surgical procedure (Fig. 2d). On hysteroscopy, the right ostium was visualised with a complete septum towards the left aspect of the cavity (Fig. 2e). Under laparoscopic control, the septum was resected by hysteroscopy using a Collin’s knife and bipolar current, midway between the fundus and the internal os, at a pre-determined depth of 48 mm. Thus, communication between the two endometrial cavities was established. The septum was resected along its entire length and the two cavities were unified. Subsequently, the patient achieved complete relief from her dysmenorrhea. Laparoscopy revealed a single fundus with evidence of endometriosis in the left adnexa. The endometrioma was enucleated and extracted.

### Case 3: Woman with two live children and dysmenorrhea

A 36-year-old patient with severe dysmenorrhea had conceived spontaneously on two occasions. She had delivered both children at full term by elective caesarean section because of breech presentation.

The patient was given analgesics for dysmenorrhea and was referred for identification of the cause of her pain. 2D US revealed a normal right hemicavity and haematometra in the left hemicavity.

**Figure 3:**
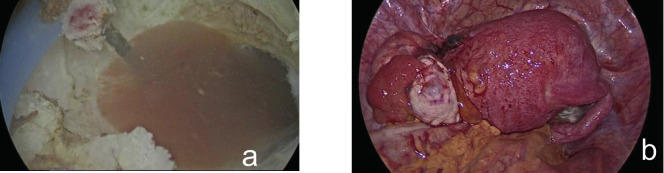
— (a,b) Laparoscopic excision of the left hemicavity and uterine reconstruction.

As the patient wished to retain her childbearing ability, we performed a laparoscopic excision of the left horn with reconstruction of the uterus (Fig. 3a,b). No further dysmenorrhea was reported on post surgical review.

### Case 4: Woman with two live children, persistent dysmenorrhea, and recurrent endometriosis

**Figure 4:**
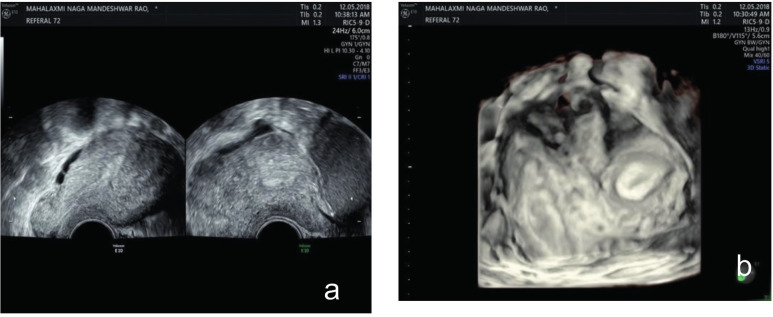
— (a, left side of the picture) Right hemicavity of the uterus with adenomyosis in the anterior wall and an endometrium of 6 mm. (a, right side of the picture) Left hemicavity of the uterus and an endometrium of 7 mm with a polyp. No communication between the left and right hemicavity. An endometrioma of 6 cm is seen in the left ovary. (b) 3D image showing normal uterine contours.

A 39-year-old patient presented with severe dysmenorrhea since menarche. Details of her obstetric history included 2 live children and a miscarriage in 2010. She also had a history of grade 4 endometriosis for which she had been operated on previously. An endometrioma on the left side had been excised in 2011. The patient was referred for routine ultrasonography with severe dysmenorrhea and recurrent endometriosis on the left side. As shown in Figure 4a, 2D US revealed a normal serosa and myometrium, and adenomyosis of the right hemicavity. The left part of the illustration shows the right hemicavity of the uterus with adenomyosis of the anterior wall and an endometrium of 6 mm. The right side of the illustration shows the left hemicavity of the uterus and an endometrium of 7 mm with a polyp. There was no communication between the left and right hemicavity. An endometrioma of 6 cm was seen in the left ovary. The contours of the uterine fundus were normal on the 3D image (Fig. 4b).

As the patient had completed her family, and due to adenomyosis with recurrent endometriosis, we performed an elective hysterectomy with left-sided salpingo-oophorectomy.

After the surgery, she reported no pain and felt satisfied.

### Case 5: Recurrent miscarriage and dysmenorrhea

A 28-year-old patient with three mid-trimester miscarriages in the 16th week of gestation was sent for ultrasonography.

**Figure 5:**
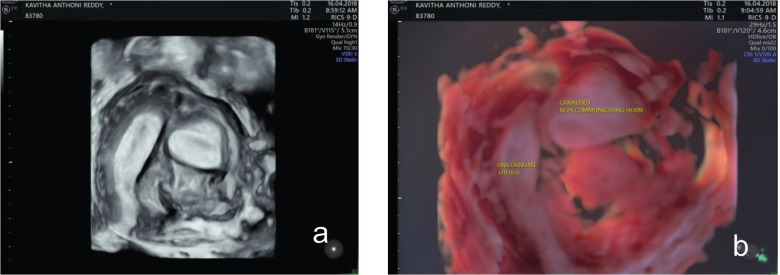
— (a) A small communicating uterine cavity on the right side and a blind uterine cavity on the left side in a 3D-rendered vaginal ultrasonic view (b) 3D live reconstruction.

A small communicating uterine cavity was seen on the right side, and a blind uterine cavity on the left side. The external shape of the uterus was normal. Figure 5a shows the 3D- rendered ultrasonographic view. Figure 5b demonstrates a 3D HD live reconstruction.

While we were planning a hysteroscopic septum resection with unification of the two uterine cavities, the patient became pregnant again. Unfortunately, due to relocation the patient was lost to follow up and there is no further information regarding pregnancy outcome.

## Discussion

### 

The female reproductive tract develops from a pair of Müllerian ducts that form the following structures: fallopian tube, uterus, cervix and the upper two-thirds of the vagina. The ovaries and lower third of the vagina have different embryological origins derived from germ cells that migrate from the primitive yolk sac and the sinovaginal bulb, respectively (Chandler et al., 2009).

The Müllerian duct is a mesoepithelial tube derived from intermediate mesoderm and composed of both epithelial and mesenchymal cells. Transcription factors and signaling molecules are necessary for the interaction between the epithelium and the mesenchyme in order to develop the Müllerian ducts. Some of these transcription factors include EMX2, HOXA13, PAX2, LIM1, and Wnt. Lim1(Lhx1) and Pax2 (Wilson et al., 2020).

The normal development of the Müllerian ducts depends on the completion of three phases: organogenesis, fusion and septal resorption. Organogenesis is characterised by the formation of both Müllerian ducts. Failure of this leads to a uterine agenesis/ hypoplasia or a unicornuate uterus. Fusion is characterised by fusion of the ducts to form the uterus. Failure of this results in a bicornuate or didelphys uterus. Septal resorption involves subsequent resorption of the central septum once the ducts have fused. Defects at this stage result in a septate or arcuate uterus (Chandler et al., 2009).

In our case series, we presented five different cases of patients with Robert`s uterus. Depending on the symptoms, the respective life situation, age and treatment options, individualised surgery was performed.

### The importance of diagnosing Robert's uterus

Robert's uterus is extremely difficult to diagnose preoperatively, and is therefore missed frequently. Diagnostic modalities may include US, hysterosalpingography (HSG), and MRI. 2D US is not very sensitive for the detection of Robert's uterus; the condition is frequently misdiagnosed as a unicornuate uterus with a non-communicating rudimentary horn, as in our above-mentioned case number one. While HSG may raise suspicion of a unicornuate uterus with a typical "banana" shape and only one fallopian tube, a septate uterus cannot be clearly visualised by HSG without imaging the external fundus. MRI is the best modality to demonstrate the uterine septum, the normal external fundal contour, haematometra and haematosalpinx, but is an expensive procedure. In cases of Robert's uterus, coronal T2-weighted MRI images are ideal for demonstrating the uterine septum dividing the endometrial cavity asymmetrically along with the blind-ending cavity and haematometra. T1-weighted images show the haematometra and haematosalpinx as bright fluid in the endometrial cavity and in a dilated fallopian tube.

3D US is equivalent to MRI and, in our opinion, even better in providing a correct diagnosis. Both diagnostic techniques require an experienced investigator.

### Diagnostic options

Gynaecological examination and 2D US are recommended for the evaluation of asymptomatic women. 3D US is recommended for the diagnosis of female genital anomalies in ‘symptomatic’ patients belonging to high-risk groups, and anomalies suspected during the routine investigation in asymptomatic women. MRI and endoscopic evaluation are recommended for patients with suspected complex anomalies or in cases of diagnostic dilemma. Adolescents with symptoms suggestive of a female genital anomaly should be thoroughly evaluated with 2D US, 3D US, MRI, and endoscopy (Grimbizis et al., 2016).

With the use of modern 3D US technology, a well-trained and dedicated ultrasonographer or gynaecologist will be able to establish Robert's uterus non-invasively and plan the correct surgical procedure accordingly. The high quality of tissue characterisation by MRI makes it an excellent diagnostic modality to confirm the diagnosis of Robert's uterus and differentiate between septate and bicornuate uteri. Many studies have shown the efficacy of MRI as well as its ability to demonstrate the endometrial cavity and uterine contours in exquisite detail. However, 3D ultrasonography is an effective alternative because it provides images of very similar quality as MRI (Deutch et al., 2008). In addition, US is well tolerated by patients, economical, and more easily available in many centres.

In 2000, Welch et al. proposed a real-time freehand 3D US system for image-guided surgery, which utilised a 5-MHz linear transducer and an optical positioner for location and orientation (Welch, 2000). At a rate of 15 frames per second, the system was able to dynamically reconstruct, update, and render 3D volumes. After performing volume measurement and visualisation in real time, a magnetic field position sensor was added to the freehand US system. Optimised sequential algorithms permitted reslicing of the 3D US volume at 10 Hz. The real-time freehand 3D US system permits semi-automatic determination of the region of interest (ROI) using a 3.5-MHz concave probe and an electromagnetic position sensor. The system was capable of rapid predetermination of the reconstruction volume and assignment of the optimal viewing direction, which achieved accurate and fast reconstruction in real time. In the absence of a predefined route, the freehand scanner should be moved over the skin surfaces at the appropriate speed that avoids significant gaps. The Voluson E10BT19 system we used for our evaluation has all of these advantages. A less than optimum US investigation may well cause the investigator to miss Robert's uterus.

### What surgical procedures are used today for the treatment of Robert's uterus?

Surgical correction is the sole effective treatment of Robert's uterus. The condition was managed via laparotomy in the early 1970s. Total resection of a hemicavity or endometrectomy of the blinded cavity, abdominal metroplasty, or a combination of these procedures with hysteroscopy/laparoscopy were used. Irreversible surgeries (resection of hemicavity/endometrectomy of the blinded cavity) were performed to improve the shape and volume of the uterine cavity. A complete endometrectomy of the blind hemicavity is suitable only in patients with no communication between the blind cavity and the ipsilateral fallopian tube. A complete endometrectomy is believed to prevent the recurrence of haematometra. In 2011, Vural et al. (2011) performed a hysterotomy incision and endometrectomy for Robert's uterus; the patient had a successful pregnancy and delivered a healthy infant by caesarean section in the 39th week of gestation. In 2017, Sardeshpande et al. (2017) performed an endometrectomy of a blind cavity in a 15-year-old patient with recurrent abdominal cramps. Li et al. (2015) reported that excision of the septum and unification of the endometrial cavity by laparotomy or hysteroscopy proved to be the correct treatment for Robert's uterus. The authors performed a septum resection with hysteroscopy under ultrasonic surveillance. The patient became pregnant and delivered a healthy child. Hysteroscopic treatment of Robert's uterus using sonohysterography without laparoscopy or laparotomy has also been reported (Ludwin et al., 2016). Under 3D sonohysterography the authors performed a hysteroscopic metroplasty guided by transrectal US, without laparotomy or laparoscopy. The outcome of the treatment was satisfactory. The patient’s menstruation ceased to be painful and she had a normal uterine cavity of 3.6 cm after a communicating hemicavity of 0.3 cm following two hysteroscopic procedures.

The term metroplasty describes a reconstructive surgery used to repair congenital anomalies of the uterus, including septate uterus and bicornuate uterus. It is an accepted method of treatment in women with recurrent miscarriages and septate uteri and it significantly improves the subsequent reproductive outcome. The incidence of spontaneous miscarriage and preterm delivery seems to decrease, whereas the incidence of term delivery rate increases (Homer et al., 2000; Kundnani et al., 2009; Venetis et al., 2014).

Hysteroscopic metroplasty for septate uterus seems to be a simple and relatively safe procedure, but nevertheless complications can occur either during the procedure (perforation of the uterus, infection, bleeding) or in subsequent pregnancy and childbirth (preterm labour, preterm delivery, higher rate of caesarean section, placenta accreta) (McCarthy et al., 2013). Nevertheless, these complications mentioned above are not significantly increased after metroplasty. One should keep in mind that the patient collective with recurrent pregnancy loss seems to be a risk factor in itself (Messerlian et al., 2013).

Although the current data does not give any definite proof of a causal relationship between septate uterus and infertility, considering the simplicity of the procedure, with low associated morbidity and the reported outcomes, the procedure should be considered in women with longstanding infertility and a septate uterus (Pai et al. 2009).

For hysteroscopic endometrial ablation or resection, surgery will be more effective if undertaken when endometrial thickness is less than 4 mm, in the immediate post-menstrual phase. However there are often difficulties in reliably arranging surgery for this time (Sowter et al., 2002). The other option is the use of hormonal agents which induce endometrial thinning or atrophy prior to surgery. A higher amenorrhoea rate after endometrium ablation can be reached by pretreatment with a GnRH analogues (e.g. Goserelin) or danazol (Romer et al., 2000). Progestogens and other GnRH analogues have also been studied although less data is available (Romer et al., 2000; Sowter et al., 2002). Endometrial thinning prior to hysteroscopic surgery leads to a clearer view, which reduces the risk of intraoperative complications such as perforation, and leads to a shorter operation time in general. Furthermore, a thin endometrium improves the intra-uterine operating environment, and reduces distension medium absorption (Sowter et al., 2002; Tan et al., 2013). This may also result in a greater improvement in long term outcomes such as menstrual loss and dysmenorrhoea. Pretreatment with GnRH analogues are generally recommended when undertaking hysteroscopic surgery on patients with a Robert`s uterus.

Our five patients were treated in accordance with the most modern standards. The entire range of treatment options were taken into account. In the fifth patient with recurrent miscarriages and no viable pregnancy, we planned the initiation of protective contraception to avoid further miscarriage. However, the patient became pregnant again.

## Conclusion

We discuss five cases of Robert's uterus, a rare type of Müllerian duct anomaly from diagnosis to treatment. Our study underlined the value of 3D vaginal US technology in detecting this congenital uterine anomaly, followed by hysteroscopy in combination with laparoscopy for final confirmation of the diagnosis. The diagnosis of Robert's uterus remains a challenge for clinicians.
